# Associations Between Intracranial Pressure Extremes and Continuous Metrics of Cerebrovascular Pressure Reactivity in Acute Traumatic Neural Injury: A Scoping Review

**DOI:** 10.1089/neur.2023.0115

**Published:** 2024-05-29

**Authors:** Kevin Y. Stein, Fiorella Amenta, Logan Froese, Alwyn Gomez, Amanjyot Singh Sainbhi, Nuray Vakitbilir, Younis Ibrahim, Abrar Islam, Tobias Bergmann, Izabella Marquez, Frederick A. Zeiler

**Affiliations:** ^1^ Biomedical Engineering, Price Faculty of Engineering, University of Manitoba, Winnipeg, Canada.; ^2^ Undergraduate Engineering, Price Faculty of Engineering, University of Manitoba, Winnipeg, Canada.; ^3^ Section of Neurosurgery, Department of Surgery, Rady Faculty of Health Sciences, University of Manitoba, Winnipeg, Canada.; ^4^ Department of Human Anatomy and Cell Science, Rady Faculty of Health Sciences, University of Manitoba, Winnipeg, Canada.; ^5^ Department of Clinical Neuroscience, Karolinska Institutet, Stockholm, Sweden.; ^6^ Division of Anaesthesia, Department of Medicine, Addenbrooke’s Hospital, University of Cambridge, Cambridge, United Kingdom.

**Keywords:** cerebrovascular reactivity, cerebral autoregulation, ICP, TBI

## Abstract

Cerebrovascular pressure reactivity plays a key role in maintaining constant cerebral blood flow. Unfortunately, this mechanism is often impaired in acute traumatic neural injury states, exposing the already injured brain to further pressure-passive insults. While there has been much work on the association between impaired cerebrovascular reactivity following moderate/severe traumatic brain injury (TBI) and worse long-term outcomes, there is yet to be a comprehensive review on the association between cerebrovascular pressure reactivity and intracranial pressure (ICP) extremes. Therefore, we conducted a systematic review of the literature for all studies presenting a quantifiable statistical association between a continuous measure of cerebrovascular pressure reactivity and ICP in a human TBI cohort. The methodology described in the Cochrane Handbook for Systematic Reviews was used. BIOSIS, Cochrane Library, EMBASE, Global Health, MEDLINE, and SCOPUS were all searched from their inceptions to March of 2023 for relevant articles. Full-length original works with a sample size of ≥10 patients with moderate/severe TBI were included in this review. Data were reported in accordance with the Preferred Reporting Items for Systematic Reviews and Meta-Analyses. A total of 16 articles were included in this review. Studies varied in population characteristics and statistical tests used. Five studies looked at transcranial Doppler-based indices and 13 looked at ICP-based indices. All but two studies were able to present a statistically significant association between cerebrovascular pressure reactivity and ICP. Based on the findings of this review, impaired reactivity seems to be associated with elevated ICP and reduced ICP waveform complexity. This relationship may allow for the calculation of patient-specific ICP thresholds, past which cerebrovascular reactivity becomes persistently deranged. However, further work is required to better understand this relationship and improve algorithmic derivation of such individualized ICP thresholds.

## Introduction

The human brain is a metabolically demanding organ, accounting for approximately 20% of the body’s total energy and oxygen consumption despite comprising only 2% of its total weight.^1^ Since the brain has limited energy stores, it requires a continuous supply of energy substrates and oxygen to satisfy its metabolic requirements. This makes cerebral tissues highly susceptible to ischemic damage, with as little as five minutes of interrupted blood flow resulting in irreversible neuronal death in several key regions of the brain.^2^ Excessive cerebral blood flow (CBF), or hyperperfusion, can also be harmful to cerebral tissues as it can result in blood–brain barrier breakdown and subsequent neurological sequelae.^1^ Therefore, maintenance of a relatively constant CBF at all times is vital for proper brain function.

The pressure driving CBF is termed cerebral perfusion pressure (CPP). This parameter represents the pressure difference between the mean arterial pressure (MAP) and the intracranial pressure (ICP); CPP = MAP − ICP. Since both MAP and ICP levels are not static, CPP frequently fluctuates. This would theoretically pose a problem for the cerebral environment, as fluctuations in CPP would expose the brain to pressure-passive changes in CBF. However, through a mechanism known as cerebrovascular pressure reactivity, cerebral vessels are able to autoregulate their tone in response to changes in CPP, allowing the brain to maintain a constant CBF despite CPP volatility.^3,4^ Unfortunately, this crucial autoregulatory mechanism is often impaired in the setting of acute mechanical neural injury, termed traumatic brain injury (TBI), and various other neurological conditions.^5^ This exposes the already injured brain to further damage from hypo- and hyperperfusion-related insults.^3,4,6^ It should be noted that the term “cerebrovascular reactivity” technically refers to vessel reactivity to various different vasodilatory and vasoconstrictive stimuli. These include myogenic/pressure, endothelial, neurogenic, metabolic, and chemo-based stimuli.^7^ However, in this article we will use the term “cerebrovascular reactivity” to refer to pressure-based reactivity.

Modern biomedical signal processing techniques now permit the continuous bedside assessment of cerebrovascular reactivity by calculating the Pearson correlation between a surrogate measure of pulsatile cerebral blood volume, such as the pulse amplitude of the ICP (AMP), and the pressure driving CBF, usually MAP or CPP.^8–12^ The resulting Pearson correlation coefficients range from −1 to +1, with negative values generally suggesting intact cerebrovascular reactivity and positive values indicating impaired reactivity.^13^ The reasoning behind this is that when cerebrovascular reactivity is intact, there should be a negative correlation between the two parameters since changes in driving pressure should be counteracted by the autoregulatory mechanism, causing small inverse changes in CBF. When impairment does exist, changes in the driving pressure would result in mirrored changes in CBF, thus producing a positive correlation between the two parameters. Various cerebrovascular reactivity indices exist; however, the commonplace of ICP monitoring in the neurocritical care setting make those based on ICP most convenient.^14^ The indices that have been studied the most include the pressure reactivity index (PRx—correlation between slow vasogenic waves of ICP and MAP), the pulse amplitude index (PAx—correlation between AMP and MAP), and RAC (the correlation (R) between AMP (A) and CPP (C)).^10,15–17^

There is currently extensive literature demonstrating that impaired cerebrovascular reactivity following moderate/severe TBI is strongly associated with poor long-term outcomes.^10,13,14,18,19^ This has led to the creation of neurocritical care consensus statements that now recommend the use of PRx for continuous cerebral autoregulatory assessment in the management of moderate/severe TBI.^20^ While the relationship between cerebrovascular reactivity and long-term outcomes has been the focus of much work, there is yet to be, to the authors’ knowledge, a comprehensive review on the association between cerebrovascular reactivity and ICP elevations, despite there being mixed literature on the subject. Such a comprehensive overview of the objective statistical relationships between ICP elevations, or extremes, and cerebrovascular reactivity is required to highlight current knowledge gaps and provide a framework for future investigations into the high-frequency physiological relationships between cerebral pressure flow, oxygenation, and nutrient delivery. It is also essential for the development of future evidence-based guideline recommendations regarding both ICP and cerebrovascular reactivity monitoring in TBI care. In this study, we conduct a systematic scoping review of the literature, compiling all quantifiable statistical associations that have been presented between measures of continuous cerebrovascular reactivity metrics, derived using modern multimodal cerebral physiological monitoring devices, and ICP in human TBI cohorts.

## Methods

A systematically conducted scoping review of the existing literature was performed following the methodology set forth by the Cochrane Handbook for Systematic Reviews.^21^ Data were reported in accordance with the Preferred Reporting Items for Systematic Reviews and Meta-Analyses Extension for Scoping Reviews (PRISMA-ScR).^22^ The completed PRISMA checklist for this systematic review can be found in Supplemental Appendix S1. The methodology and strategy used align with that of previous scoping reviews conducted by our research team.^23–26^ Review objectives and search strategy were formulated by the primary (K.Y.S.) and senior (F.A.Z.) authors.

### Search question and inclusion/exclusion criteria

In this scoping review of the literature, the following question was evaluated: What is the association between continuous metrics of cerebrovascular pressure reactivity and ICP elevations/extremes in the TBI population?

Inclusion criteria were as follows: original peer-reviewed full-length research articles, human moderate/severe TBI (Glasgow Coma Scale [GCS] ≤12) cohort with a sample size of ≥10, use of invasive ICP monitoring, and presentation of an objective quantifiable statistical association between cerebrovascular pressure reactivity and ICP. Non-original, non-relevant, animal-based, and non-English language studies were excluded from this review.

### Search strategy

Six independent databases were searched from their inceptions up until March of 2023 using the search strategy outlined in Supplemental Appendix S2. These databases included BIOSIS, Cochrane Library, EMBASE, Global Health, MEDLINE, and SCOPUS. Articles found through this search were compiled and deduplicated prior to being filtered for inclusion.

### Study selection

All articles went through a meticulous two-step review using two reviewers (K.Y.S. and F.A.). In the first filtering phase, the reviewers independently screened the titles and abstracts of all unique articles identified during the search process in order to exclude those that fulfilled any of the exclusion criteria. Next, the full-length documents of all articles that were deemed possibly eligible during the first phase were assessed to confirm that they meet all inclusion criteria. Any discrepancies between the two reviewers during the filtering process were resolved by the senior author (F.A.Z.). Finally, the reference lists of all articles that reached full-text examination were assessed for any potentially relevant articles that were missed.

### Data collection

The following information, when available, was collected from each of the included studies and summarized in Table 1: country of study, sample size, population description, age, sex, GCS, mean ICP, Marshall computed tomography (CT) grade, pupillary response, number with traumatic subarachnoid hemorrhage (tSAH), number with epidural hematoma (EDH), number with subdural hematoma (SDH), and primary objective of study. All statistical associations between cerebrovascular reactivity and ICP that were found in the included studies are presented in Table 2.

**Table 1.** Overview of Included Articles

Source	Country	Sample size (Patients with TBI)	Population	Age	Sex (% male)	Admission GCS score	Mean ICP (mmHg)	Marshall CT grade	Pupillary response	tSAH, EDH, SDH	Primary objective of study
Consonni et al.^27^	Italy	16 (26 total, 10 non-TBI)	Mixed Population—Majority TBI	Mean (± SD): 47 ± 17	69.2%	Median: 8	–	–	–	–	To evaluate whether daily bedside assessment of cerebral autoregulation is feasible and whether such data provide any clinical utility.
Czosnyka et al.^28^	UK	82	TBI	Mean: 36 Range: 6–75	67.1%	Mean: 6 Range: 3–13	–	–	–	– 20.3% 13.5%	To evaluate whether it is possible to continuously assess autoregulatory reserve by comparing changes in FVm and FVs with spontaneous changes in CPP.
Czosnyka et al.^16^	UK	82	TBI	Mean: 36 Range: 6–75	67.1%	Mean: 6 Range: 3–13	–	–	–	–	To evaluate whether slow waves of ABP and ICP can be used to derive a metric that reflects vascular reactivity to ABP change.
Czosnyka et al.^29^	UK	187	TBI	Mean: 36 Range: 6–75	76.5%	Median: 6 Range: 3–13	–	–	–	25% 10% 31%	To evaluate the relationship between cerebral autoregulation, ICP, ABP, and CPP after head injury.
Donnelly et al.^30^	UK	24 (33 total, 9 had no PRx)	TBI	Mean (± SD): 30.3 ± 12.5	78.8%	84.8% ≤ 8	Mean (± SD): 25.6 ± 8.71	–	–	–	To evaluate the cerebral physiological response to severe and sustained intracranial hypertension.
Eide et al.^31^	UK	76	TBI	Median: 27.5 Range: 3–71.5	76.3%	Median: 5 Range: 3–11	Median: 18.0 Range: 3.1–73.7	–	–	–	To evaluate whether the pulse amplitudes of ICP and arterial pressure are interrelated and whether correlations between the two are associated with cerebral autoregulatory indices.
Gao et al.^32^	UK	174	TBI	Mean (± SD): 37.8 ± 15.0	75.3%	–	–	–	–	–	To evaluate whether early signal complexity measures are associated with neurological outcome.
Lang et al.^33^	Germany	40	TBI	Mean (± SD): 40 ± 16	80.0%	Mean: 8	–	–	–	– 15% 57.5%	To validate PRx and evaluate its relationship with cerebral blood flow velocity and cerebral autoregulatory capacity.
Lewis et al.^34^	UK	21	TBI	Mean: 24 Range: 17–71	81.0%	Median: 4 Range: 3–11	–	–	–	–	To compare the behavior of Fix and Mx during ICP plateau waves and demonstrate the behavior of Fix during periods of reduced CPP.
Liu et al.^35^	UK	515	TBI	Mean (± SD): 38.4 ± 16	74.8%	Median: 7 IQR: 3–9	Mean (± SD): 16.2 ± 12.2	–	–	–	To utilize wavelet transform analysis for the assessment of cerebrovascular pressure reactivity and compare its performance with PRx.
Lu et al.^36^	UK	290	TBI	Mean (± SD): 38 ± 16.5	77.9%	Median: 6 IQR: 3–9	–	–	–	–	To evaluate the complexity of ICP and its associations with outcome in TBI using multiscale entropy.
Sánchez-Porras et al.^37^	Germany	29	TBI	Mean: 37.2 Range: 5–75	89.6%	–	–	–	–	–	To evaluate the prognostic value of L-PRx in TBI.
Smith et al.^38^	South Africa	196	Pediatric TBI	Median: 6.7 IQR: 4.3–9.1	66.8%	–	Median: 12.4 IQR: 9.6–15.3	–	–	–	To evaluate PRx and its associations with physiological variables and outcome in pediatric severe TBI.
Steiner et al.^39^	UK	114	TBI	Mean (± SD): 34 ± 16	84.2%	Mean (± SD): 6.6 ± 2.8	Mean (± SD): 18 ± 9	–	–	–	To define optimal CPP in head injured patients using PRx.
Wettervik et al.^40^	Sweden	120	TBI	Mean (± SD): 43 ± 20	75%	–	Mean (± SD): 13 ± 10	Median: 2 IQR: 2–5	Abnormal: 22%	–	To evaluate the utility of mild hyperventilation and its association with pressure autoregulation and outcome.
Zeiler et al.^41^	EU	185	TBI	Median: 51 IQR: 31–62.3	76.2%	Median: 6 IQR: 3–7	–	Median: 3 IQR: 2–6	BR: 73.0% UU: 8.1% BU:18.9%	61.6% 19.5% –	To evaluate the physiological effects of elevated ICP, using a multicenter database, by comparing those with a mean ICP < 15 mmHg and those with a mean ICP > 20 mmHg.

ABP, arterial blood pressure; BR, bilaterally reactive; BU, bilaterally unreactive; CPP, cerebral perfusion pressure; CT, computed tomography; EDH, epidural hematoma; EU, European Union; Fix, Flow-ICP index; FV, transcranial Doppler flow velocity; FVm, mean FV; FVs, systolic FV; GCS, Glasgow Coma Scale; ICP, intracranial pressure; IQR, interquartile range; L-PRx, low-frequency pressure reactivity index; Mx, mean flow index; NICU, neurointensive care unit; PRx, pressure reactivity index; SD, standard deviation; SDH, subdural hematoma; TBI, traumatic brain injury; tSAH, traumatic subarachnoid hemorrhage; UK, United Kingdom; UU, unilaterally unreactive.

**Table 2.** Findings regarding Associations between ICP Extremes and Multimodal Continuous Cerebrovascular Reactivity Metrics

Source	Sample size (Patients with TBI)	Measure correlated with ICP threshold	Findings					
Consonni et al.^27^	16 (26 total, 10 non-TBI)	PRx	Independent two-tailed *t*-test:					
				Intact (PRx < 0)		Null (0 < PRx < 0.2)		Deranged (PRx > 0.2)
			ICP max (mmHg) [median (IQR)]	25 ± 10.2		22 ± 9.0		30 ± 18.3
			ICP min (mmHg) [median (IQR)]	10 ± 5.3		6 ± 4.8		12 ± 15.4
				Intact vs Null		Intact vs Deranged		Null vs Deranged
			ICP max (*p* value)	**0.04**		**0.03**		**<0.01**
			ICP min (*p* value)	**<0.01**		0.23		**<0.01**
Czosnyka et al.^28^	82	Mx and Sx	Spearman’s correlation between mean ICP and …					
			Mx:		*r* = 0.456		***p* < 0.0001**	
			Sx:		*r* = 0.340		***p* < 0.003**	
Czosnyka et al.^16^	82	PRx and Mx	Spearman’s correlation between mean ICP and …					
			PRx:		*r* = 0.36		***p* < 0.001**	
			Mx:		*r* = 0.455		***p* < 0.0001**	
			One way ANOVA test between PRx and ICP presented a statistically uneven distribution (***p* < 0.01**), with a general positive trend.					
Czosnyka et al.^29^	187	Mx	Mann–Whitney U test:					
				Mean ICP < 25 mmHg		Mean ICP ≥ 25 mmHg		*p* value
			Mx [mean (SD)]	−0.05		0.22 ± 0.35		**0.000006**
Donnelly et al.^30^	24 (33 total, 9 had no PRx)	PRx	Mann–Whitney U test:					
				Mean ICP (mmHg)				
				Baseline (0–25)		Elevated (25–50)		Severe (50–150)
			PRx [mean (SD)]	0.06 (0.26)		0.21 (0.30)		0.57 (0.24)

				Base vs Elevated		Elevated vs Severe		
			*p* value	**0.01**		**<0.001**		*p* value
			LOWESS curve with 95% CI demonstrated a general positive correlation between ICP and PRx.					
Eide et al.^31^	76	PRx and Mx	Multiple linear regression analysis: Greater mean ICP was associated with greater PRx (***p* = 0.001**) and Mx (***p* = 0.003**)					
Gao et al.^32^	174	PRx	Approximate entropy of ICP (ApEn-ICP), a metric of ICP signal complexity, was inversely correlated with PRx (*r* = −0.39, ***p* < 0.000001**).					
Lang et al.^33^	40	PRx	Independent two-tailed *t*-test:					
				Mean PRx < 0.3		Mean PRx > 0.3		
			Mean ICP (mmHg) [mean (SD)]	14 ± 7		16 ± 7		*p* > 0.001 (insignificant)
Lewis et al.^34^	21	Mx, Fix	Spearman’s correlation coefficient between mean ICP and …					
			Mx:		*r* = 0.70		***p* < 0.00001**	
			Fix:		*r* = −0.67		***p* < 0.00001**	
Liu et al.^35^	515	PRx, wPRx	Spearman’s correlation coefficient between mean ICP and …					
			PRx:		*r* = 0.11		***p* = 0.029**	
			wPRx:		*r* = 0.32		***p* < 0.001**	
Lu et al.^36^	290	PRx	Spearman’s correlation coefficient between the signal complexity index of ICP and PRx: *r* = −0.25, ***p* = < 0.0001**					
Sánchez-Porras et al.^37^	29	L-PRx	Spearman’s correlation coefficient between mean ICP and L-PRx: *r* = 0.467, ***p* = 0.011**					
Smith et al.^38^	196	PRx	Spearman’s correlation coefficient between mean ICP and PRx: *r* = 0.44, ***p* < 0.001**					
Steiner et al.^39^	114	PRx	Spearman’s correlation coefficient between mean ICP and PRx: *r* = −0.068, *p* = 0.475					
Wettervik et al.^40^	120	PRx55-15	Multiple linear regression analysis: Greater ICP was associated with greater PRx55-15 (***p* = 0.006**)					
Zeiler et al.^41^	185	PRx, PAx, RAC	Mann–Whitney U test:					
				Mean ICP < 15 mmHg		Mean ICP > 20 mmHg		
			Mean PRx [median (IQR)]	−0.002 (−0.118–0.014)		0.206 (−0.009–0.582)		***p* = 0.0006**
			Mean PAx [median (IQR)]	−0.090 (−0.208–0.095)		0.151 (−0.090–0.376)		***p* = 0.0001**
			Mean RAC [median (IQR)]	−0.384 (−0.554–−0.176)		−0.181 (−0.382–0.145)		***p* = 0.0030**
			% time above threshold…					
			PRx > 0 [median (IQR)]	47.8 (22.6–64.3)		72.4 (47.1–90.2)		***p* = 0.0009**
			PRx > +0.25 [median (IQR)]	23.7 (14.0–38.0)		46.1 (26.2–82.1)		***p* = 0.0003**
			PRx > +0.35 [median (IQR)]	17.0 (9.70–28.0)		36.4 (18.3–77.9)		***p* = 0.0003**
			PAx > 0 [median (IQR)]	39.6 (25.6–59.9)		66.4 (41.4–79.6)		***p* = 0.0002**
			PAx > +0.25 [median (IQR)]	16.0 (8.80–32.1)		36.4 (19.2–62.6)		***p* = <0.0001**
			RAC >−0.10 [median (IQR)]	20.9 (9.20–40.4)		39.4 (18.1–75.1)		***p* = 0.0030**
			RAC >−0.05 [median (IQR)]	17.6 (7.80–35.4)		35.3 (15.9–71.2)		***p* = 0.0020**
			Mean hourly dose above threshold …					
			PRx > 0 [median (IQR)]	7.5 (4.9–11.3)		12.5 (6.2–26.9)		***p* = 0.0030**
			PRx > +0.25 [median (IQR)]	2.9 (1.7–4.4)		6.0 (2.5–16.1)		***p* = 0.0006**
			PRx > +0.35 [median (IQR)]	1.8 (1.1–2.9)		4.1 (1.6–12.3)		***p* = 0.0004**
			PAx > 0 [median (IQR)]	5.0 (2.9–9.5)		10.3 (5.9–17.3)		***p* = 0.0003**
			PAx > +0.25 [median (IQR)]	1.6 (0.7–3.4)		4.3 (2.3–8.6)		***p* = <0.0001**
			RAC >−0.10 [median (IQR)]	2.6 (1.1–5.4)		5.8 (2.1–15.8)		***p* = 0.0010**
			RAC >−0.05 [median (IQR)]	2.0 (0.8–4.0)		4.9 (1.8–13.7)		***p* = 0.0007**

^*^ These studies looked at ICP signal complexity rather than ICP itself.

AMP, pulse amplitude of ICP; ANOVA, analysis of variance; CI, confidence interval; CPP, cerebral perfusion pressure; Fix, Flow-ICP index; FV, transcranial Doppler flow velocity; ICP, intracranial pressure; IQR, interquartile range; LOWESS, locally estimated scatterplot smoothing; L-PRx, low-frequency pressure reactivity index; mmHg, millimeters of mercury; Mx, mean flow index; PAx, pulse amplitude index; PRx, pressure reactivity index; PRx55-15, pressure reactivity index with 15–55 second period bandpass filter; RAC, correlation (R) between AMP (A) and CPP (C); Sx, systolic index; TBI, traumatic brain injury; wPRx, wavelet pressure reactivity index.

### Statistical analysis

Considering the highly heterogeneous nature of the study designs and results of cerebral physiology literature, no formal meta-analysis was performed.

### Bias assessment

Given that the primary aim of this review was to conduct a comprehensive scoping review of the literature, a formal bias assessment was not warranted.

## Results

### Search strategy results

**FIG. 1.**
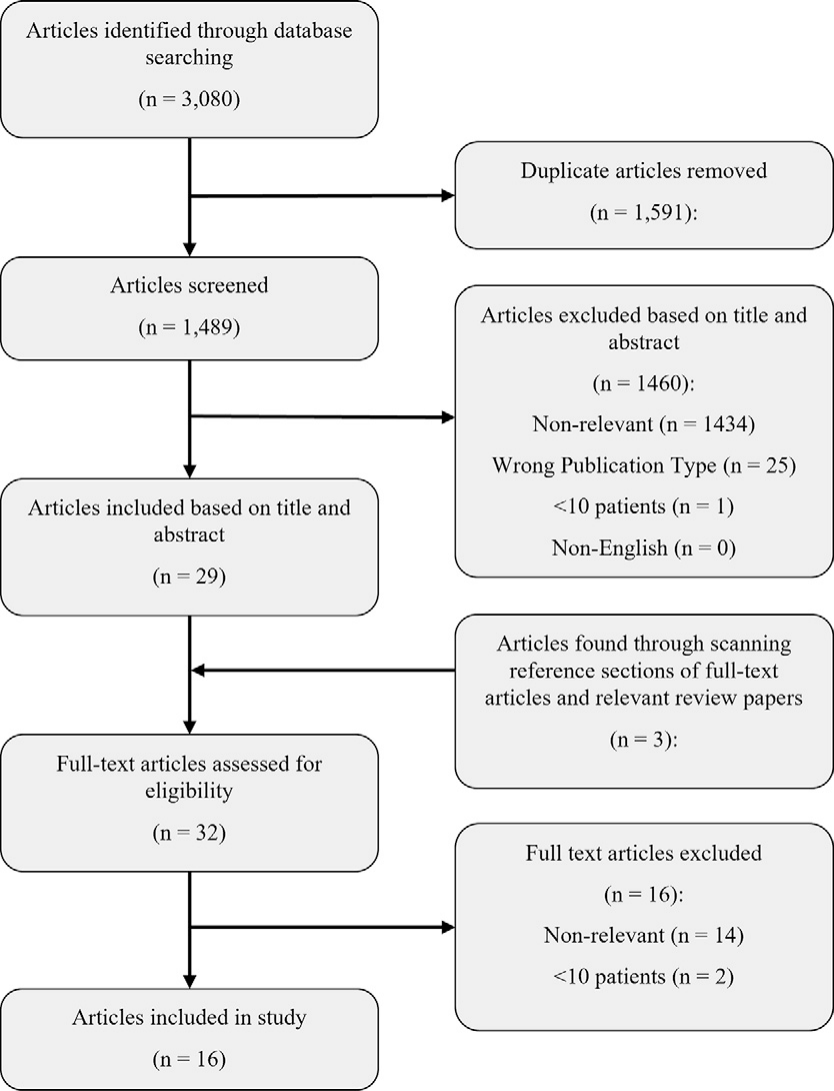
PRISMA flow diagram. PRISMA, Preferred Reporting Items for Systematic Reviews and Meta-analyses.

A PRISMA flow diagram summarizing the search results, as well as the number of articles excluded at each filtering stage, is presented in Figure 1. The search strategy returned a total of 3080 articles. Following deduplication, 1591 articles were removed, leaving 1489 unique for article review. The titles and abstracts of these articles were then screened using the inclusion and exclusion criteria, resulting in 29 articles being deemed eligible for full-text review. Exclusion criteria were applied in the following order: non-relevant, wrong study design, sample size <10, and non-English language. Of the 1460 articles that were excluded, 1434 were excluded for being non-relevant, 25 were excluded for not being full-length original works, and 1 was excluded for having a sample size of <10. The reference sections of the 29 eligible articles, and 1 relevant review article, were then screened for any relevant articles that were missed. This resulted in 3 more articles being added for full-text review, bringing the total up to 32 articles.

Following full-text review, only 16 articles were found to fully satisfy all of the inclusion criteria. Of the 16 articles that were excluded, 14 were excluded for being non-relevant, while 2 were excluded for having sample sizes of <10 patients with TBI. Study designs and basic demographic data for the 16 included articles are summarized in Table 1. There was a high level of variability in terms of demographics reported among the articles. In addition, the type of statistics used to report results was highly heterogeneous. The majority of the included articles studied general moderate/severe TBI cohorts; however, one study restricted their investigation to a cohort of pediatric (≤14 years of age) patients. Except for 1 study that was based out of South Africa, all studies originated in Europe, with 10 coming from a single center in Cambridge, UK.

The relevant findings from each of the 16 included studies are summarized in Table 2. Each study presented a quantifiable statistical association between a continuous cerebrovascular reactivity index and ICP; however, two studies looked at ICP signal complexity, the degree of ICP waveform irregularity, rather than ICP level itself.^32,36^ One study utilized a pediatric patient cohort, while all other studies used adult cohorts. A range of cerebrovascular reactivity indices were used among the studies, all of which are summarized in Table 3. Five studies investigated transcranial doppler (TCD) based indices, which included the mean flow index (Mx—correlation between TCD flow velocity and CPP), the systolic index (Sx—correlation between systolic TCD flow velocity and CPP), and the flow-ICP index (Fix—correlation between ICP and CBF velocity). Thirteen studies investigated ICP-based indices, which included PRx, PAx, RAC, low-frequency PRx (L-PRx), wavelet PRx (wPRx), and PRx focused on the vasogenic frequency range associated with cerebral autoregulation (PRx55-15). Various different statistical methods were used to demonstrate a relationship between cerebrovascular reactivity and ICP.

**Table 3.** Cerebrovascular Reactivity Indices Used among the Included Articles

Index	Derivation
Fix	Pearson’s correlation between 30 consecutive 10-second averages of ICP and CBFV, updating every minute.
L-PRx	Pearson’s correlation between 20 consecutive 1-minute averages of ICP and ABP, updating every minute.
Mx	Pearson’s correlation between 30 consecutive 10-second averages of CPP and FV, updating every minute.^a^
PAx	Pearson’s correlation between 30 consecutive 10-second averages of AMP and ABP, updating every minute.
PRx	Pearson’s correlation between 30 consecutive 10-second averages of ICP and ABP, updating every minute.^a^
PRx55-15	Pearson’s correlation between 30 consecutive 10-second averages of ICP and ABP, updating every minute, with a bandpass filter for oscillations with periods of 15–55 seconds applied.
RAC	Pearson’s correlation between 30 consecutive 10-second averages of AMP and CPP, updating every minute.
Sx	Pearson’s correlation between 36 consecutive 5-second averages of CPP and systolic FV, updating every 3 minutes.
wPRx	Cosine of the wavelet transform phase shift between ICP and ABP, in the frequency of 0.0067 Hz to 0.05 Hz, calculated using a 500-second window, updating every 10 seconds.

^a^ Variability among included studies in regard to window size, update frequency, and number of data points used.

ABP, arterial blood pressure; AMP, pulse amplitude of ICP; CBFV, cerebral blood flow velocity; CPP, cerebral perfusion pressure; Fix, Flow-ICP index; FV, transcranial Doppler flow velocity; ICP, intracranial pressure; L-PRx, low-frequency pressure reactivity index; Mx, mean flow index; PAx, pulse amplitude index; PRx, pressure reactivity index; PRx55-15, pressure reactivity index with 15–55 second period bandpass filter; RAC, correlation (R) between AMP (A) and CPP (C); Sx, systolic index; wPRx, wavelet pressure reactivity index.

### TCD-based indices

Among the five studies that investigated TCD-based continuous cerebrovascular reactivity indices, all five looked at Mx, while both Sx and Fix were only looked at in single studies. A 2001 study by Czosnyka and colleagues dichotomized patients into those with a mean ICP < 25 mmHg and those with a mean ICP ≥ 25 mmHg and evaluated differences between the cohorts using Mann–Whitney U testing.^29^ They found that Mx was significantly greater in the elevated ICP group (*p* = 0.000006). In a 1996 study by Czosnyka and colleagues, Mx and Sx were both found to have statistically significant correlations with mean ICP, producing Spearman’s correlation coefficients of 0.456 (*p* < 0.0001) and 0.340 (*p* < 0.003), respectively.^28^ In another study by Czosnyka and colleagues, published 1 year later, they once again found that Mx was correlated with mean ICP (*r* = 0.455, *p* < 0.0001).^16^ However, it should be noted that this study used the exact same dataset as the previous one. In a 2014 study by Lewis and colleagues, Mx and Fix were found to be statistically correlated with ICP, producing Spearman’s correlation coefficients of 0.70 (*p* < 0.00001) and −0.67 (*p* < 0.00001).^34^ Finally, using multiple linear regression analysis, Eide and colleagues found that greater mean ICP was associated with greater Mx (*p* = 0.003).^31^

### ICP-based indices

Among the 13 studies looking at ICP-based continuous cerebrovascular reactivity indices, all but one looked at high-frequency metrics. Donnelly and colleagues separated patients based on mean ICP into three groups: *baseline* (0–25 mmHg), *elevated* (25–50 mmHg), and *severe* (50–150 mmHg).^30^ Upon Mann–Whitney U testing, PRx was found to be significantly greater with each increasing ICP category, producing *p*-values of 0.01 and <0.001 for *baseline* versus *elevated* and *elevated* versus *severe*, respectively. The authors also presented a locally estimated scatterplot smoothing (LOWESS) curve with 95% confidence intervals which exhibited a general positive correlation between PRx and ICP. However, the curve turns downwards at very high values of ICP and turns upwards at very low levels, producing a somewhat sinusoidally shaped curve. Zeiler and colleagues dichotomized patients into those with a mean ICP < 15 mmHg and those with a mean ICP > 20 mmHg.^41^ They then compared various cerebrovascular reactivity measures between the two groups. PRx, PAx, and RAC were all found to be greater in the elevated ICP group, with *p*-values of 0.0006, 0.0001, and 0.0030, respectively. They also looked at % times, as well as mean hourly doses, above various thresholds; PRx > 0, PRx > +0.25, PRx > +0.35, PAx > 0, PAx > +0.25, RAC > −0.10, and RAC > −0.05. All % times and mean hourly doses above thresholds were significantly higher in the elevated ICP group (all *p*-values < 0.01). Two studies opted to group patients based on cerebrovascular reactivity (mean PRx for both) instead of ICP and used independent two-tail *t*-tests to find whether there were significant differences in ICP between the groups.^27,33^ Consonni and colleagues divided patients into three groups: *Intact* (PRx < 0), *Null* (0 < PRx < 0.2), and *Deranged* (PRx > 0.2).^27^ They then compared both ICP max and ICP min between the three groups. The *Deranged* group had the greatest ICP max and min values, while the *Null* group had the lowest values. All *t*-tests achieved statistical significance (*p* < 0.05), except for the test between the *Intact* and *Deranged* groups for ICP min (*p* = 0.23). Lang and colleagues chose to split patients into only two groups, those with a mean PRx < 0.3 versus those with a mean PRx > 0.3.^33^ When a *t*-test was performed, it failed to find a statistically significant difference in mean ICP between the groups.

In a 1997 study by Czosnyka and colleagues, PRx was found to be correlated with ICP, producing a Spearman’s correlation coefficient of 0.36 (*p* < 0.0001).^16^ The authors also graphed the results of a one-way analysis of variance (ANOVA) test between PRx and ICP. This graph exhibited a similar trend as the LOWESS curve seen in the study by Donnelly and colleagues,^30^ where a general positive trend exists except at low and extremely high levels of ICP. Liu and colleagues found that both PRx and the wavelet pressure reactivity index (wPRx) were correlated with ICP, producing correlation coefficients of 0.11 (*p* = 0.029) and 0.32 (*p* < 0.001), respectively.^35^ Smith and colleagues demonstrated a correlation between PRx and ICP, presenting a correlation coefficient of 0.44 (*p* < 0.001).^38^ However, Steiner and colleagues failed to demonstrate a statistically significant correlation between the two parameters (*p* = 0.475).^39^ Gao and colleagues looked at the approximate entropy of ICP (ApEn-ICP), a metric of ICP signal complexity, and found that it was inversely corelated with PRx (*r* = −0.39, *p* < 0.000001).^32^ Lu and colleagues also investigated ICP signal complexity, finding a similar result with a Spearman’s correlation coefficient of −0.25 (*p* < 0.0001).^36^ Finally, using multiple linear regression analysis,Wettervik and colleagues found that greater ICP was associated with greater PRx55-15 (*p* = 0.006).^40^

Only one study looked at a low-frequency ICP-based index. In this study, Sánchez-Porras and colleagues found L-PRx to be correlated with ICP and presented a Spearman’s correlation coefficient of 0.467 (*p* = 0.011).^37^

## Discussion

In this review, we systematically scoured the existing literature for all studies that present a quantifiable statistical association between continuous metrics of cerebrovascular pressure reactivity and ICP elevations in a TBI cohort. Through this process, we were able to uncover multiple interesting findings. However, it should be noted that there was a significant amount of heterogeneity in terms of statistical tests and cerebrovascular reactivity indices used among the included studies, limiting our ability to compare their results. Overall, the literature seems to suggest that a relationship between cerebrovascular reactivity and ICP exists, where impaired reactivity is associated with increased ICP. Of the 16 included studies, only 2 failed to demonstrate a statistically significant association between the two parameters.^33,39^ However, it is important to acknowledge that reaching statistical significance does not speak on the strength of the association. For example, the two studies using multiple linear regression analysis demonstrated statistically significant associations between cerebrovascular reactivity and ICP but did not comment on the strengths of these relationships.^31,40^

In regard to the TCD-based indices, all relevant studies demonstrated an association between impaired cerebrovascular reactivity and elevated ICP. While the results from the studies that used Mann–Whitney U testing and multiple linear regression analysis do not allow us to comment on the strength of this relationship, the three studies that presented Spearman’s correlation coefficients provide us some valuable insight into its strength. The three correlation coefficients that were found for Mx ranged from 0.455 to 0.70, indicating a fairly strong correlation with ICP.^16,28,34^ However, it should be noted that two of the three studies utilized the same dataset,^16,28^ and the third had a sample size of only 21.^34^ The one correlation coefficient presented for Sx was weaker, at only 0.32,^28^ indicating that it may not be as strongly correlated with ICP as Mx is. Interestingly, Fix was found to be negatively correlated with ICP; however, this can be explained by the fact that, in contrast with other indices, lower Fix indicates more impaired cerebrovascular reactivity.^34^

Looking at the studies that presented Spearmen’s correlations between an ICP-based cerebrovascular reactivity index and ICP, we saw that all but one were able to reach statistical significance.^16,35,37–39^ The three statistically significant correlation coefficients found for PRx ranged from 0.11 to 0.44, suggesting a moderately strong positive correlation with ICP.^16,35,38^ The correlation coefficient for wPRx also fell within this range.^35^ Interestingly, the correlation coefficient presented for L-PRx was larger, at 0.467, suggesting that the low-frequency method of derivation may result in a stronger association with ICP. It is also interesting to see that, overall, the TCD-based indices demonstrated stronger correlations with ICP than PRx, considering the heavy focus on this index in current cerebral physiological research.

Among the studies that grouped patients based on mean ICP and used Mann–Whitney U testing, we saw that elevated ICP was consistently statistically associated with more impaired cerebrovascular reactivity.^29,30,41^ Differences in means between ICP groupings were quite sizable as well, with the smallest being a ΔPRx of 0.15 between the *baseline* and *elevated* groups in the study by Donnelly and colleagues.^30^ However, it should be noted that this study had a significantly smaller sample size (*n* = 24) than the other two studies. Interestingly, Zeiler and colleagues also included % time and mean hourly doses above literature guided cerebrovascular reactivity thresholds to their analysis, finding that increased values of these parameters were also associated with higher ICP.^41^ It seems that PAx potentially has a stronger association with ICP than PRx and RAC considering that it, and its % time and mean hourly doses above thresholds, consistently had the lowest *p*-values and greatest differences between the normal and elevated ICP groups. However, it is difficult to make any conclusive statements about this without further evidence.

The results found in three studies mentioned above somewhat conflict with those found in the studies that grouped patients based on cerebrovascular reactivity and used independent two-tailed *t*-tests instead.^27,33^ The study by Lang and colleagues failed to find any statistically significant difference between *Intact* and *Deranged* PRx groups.^33^ The findings of this study are quite surprising as one would except its results to mirror those found in the previous three studies. Consonni and colleagues were able to find a statistically significant difference between the *Intact* and *Deranged* groups for ICP max, but not for ICP min.^27^ This lack of a statistically significant difference in ICP min may be explained by the fact that PRx tends to function poorly at low ICP values.^42^ This is because PRx derivation uses ICP as a surrogate for cerebral blood volume, and that at low levels, ICP does not accurately reflect changes in cerebral blood volume. Another interesting finding here is that the *Null* group had lower ICP max and ICP min values than both the *Intact* and *Deranged* groups despite having an intermediate mean PRx range (0 < PRx < 0.2).^27^ This may suggest that the relationship between cerebrovascular reactivity and ICP is not linear, but rather curved. However, it is important to note that both of these studies had sample sizes well below the recommended minimum sample size for cerebral physiological research.^43^ We must therefore be cautious in interpreting their findings.

The two studies that looked at ICP signal complexity both found statistically significant negative correlations with PRx, producing moderate coefficients of -0.39 and -0.25.^32,36^ This falls well in line with existing literature that has suggested that decreased ICP signal complexity is associated with intracranial hypertension and worse outcomes.^36,44,45^

### Limitations of the literature

Although the existing literature presented multiple interesting findings, there are some important limitations that must be highlighted. First, the relevant studies were somewhat heterogeneous in regard to their statistical methodologies. This hindered our ability to compare results fully, as we could compare only studies that used the same statistical test. Population characteristics also varied among the studies. Although all studies utilized a TBI dataset, minimum age and injury severity of patients were variable. Next, a substantial portion of the included studies had small sample sizes. Four of the studies had sample sizes <30,^27,30,34,37^ and another four had sample sizes <85.^16,28,31,33^ This puts half of the included studies well under the recommended minimum of 100 for cerebral physiological research,^43^ bringing the reliability of their results into question. Finally, the study populations were predominantly European, with only one study originating from outside of Europe.^38^ Furthermore, 10 of the 16 included studies originated from the same single center in Cambridge, UK.^16,28–32,34–36,39^ This may limit the external generalizability of our findings to the broader population.

### Limitations of the study

Despite the rigorous and systemic manner with which this review was conducted, there are a couple of noteworthy limitations of this study that must be addressed. First, this study included only English-language articles. Although no studies were excluded on the basis of being non-English during the filtering stages, it is possible that some relevant articles were not picked up during the database search due to not being in English. Second, this study only considered studies that investigated TBI populations. Inclusion of studies looking at healthy volunteers or other neurologically ill populations, including animal-based literature, could have possibly provided us a better picture of the relationship between the cerebrovascular reactivity and ICP. However, we chose to restrict our review since a better understanding of the relationship between cerebrovascular reactivity and ICP has potential to improve moderate/severe TBI management.

### Future directions

Due to the limitations listed above, a large multicentered study investigating the relationships between the various cerebrovascular reactivity indices and ICP using time-series statistical methodologies which evaluate high-frequency multivariate behaviors could prove to be beneficial, as this could help answer any remaining questions about this important relationship. Moving forward, it may be possible to use this relationship between ICP and cerebrovascular reactivity to identify patient-specific ICP thresholds, past which cerebrovascular reactivity becomes persistently deranged. Preliminary work demonstrating the feasibility of determining such an individualized ICP (iICP) threshold has already been done.^46,47^ Lazaridis and colleagues first introduced this concept in 2014, when they plotted ICP against PRx and visually identified the ICP value past which PRx surpasses a threshold of +0.20.^46^ They were able to identify such an iICP in approximately 68% of patients. In 2021, Zeiler and colleagues were able to semi-automate this method and demonstrated that dose-time above iICP was more closely associated with outcome than dose-time above guideline-based ICP thresholds.^47^ However, this concept is far from clinically applicable in its current form as both studies utilized entire recording periods to derive iICP. In addition, both works have used PRx exclusively. Further work is therefore needed to facilitate continuously updating iICP at patient bedside, improve % yield, compare performances of various cerebrovascular reactivity indices and thresholds for iICP derivation, and optimize outcome prognostication accuracy. Such work is currently being undertaken by our laboratory group.

## Conclusion

Based on the findings of this systematically conducted scoping review, we conclude that there exists a moderately strong relationship between continuous cerebrovascular reactivity indices and ICP elevations, with impaired reactivity being associated with higher ICP and lower ICP signal complexity. This relationship has been shown previously to allow for the development of patient-specific ICP thresholds past which cerebrovascular reactivity becomes persistently deranged. However, further work is required to better understand the higher temporal relationships between ICP and cerebrovascular reactivity and develop clinically applicable iICP algorithms.

## Supplementary Material

Supplemental Appendix S1

## Supplementary Material

Supplemental Appendix S1

## Abbreviations Used

ABP: arterial blood pressure
AMP: pulse amplitude of ICP
ANOVA: analysis of variance
ApEn-ICP: approximate entropy of ICP
BR: bilaterally reactive
BU: bilaterally unreactive
CBF: cerebral blood flow
CBFV: cerebral blood flow velocity
CI: confidence interval
CPP: cerebral perfusion pressure
CT: computed tomography
EDH: epidural hematoma
EU: European Union
Fix: flow-ICP index
FV: TCD flow velocity
FVm: mean FV
FVs: systolic FV
GCS: Glasgow Coma Scale
ICP: intracranial pressure
iICP: individualized ICP threshold
IQR: interquartile range
LOWESS: locally estimated scatterplot smoothing
L-PRx: low-frequency
PRxMAP: mean arterial pressure
mmHg: millimeters of mercury
Mx: mean flow index
NICU: neurointensive care unit
PAx: pulse amplitude index
PRISMA-ScR: Preferred Reporting Items for Systematic Reviews and Meta-Analyses Extension for Scoping Reviews
PRx: pressure reactivity index
PRx55-15: PRx with 15-55 second period bandpass filter
RAC: correlation (R) between AMP (A) and CPP (C)
SD: standard deviation
SDH: subdural hematoma
Sx: systolic index
TBI: traumatic brain injury
TCD: transcranial doppler
tSAH: traumatic subarachnoid hemorrhage
UK: United Kingdom
UU: unilaterally unreactive
wPRx: wavelet PRx
