# Supplementary material for: Associations Between Intracranial Pressure Extremes and Continuous Metrics of Cerebrovascular Pressure Reactivity in Acute Traumatic Neural Injury: A Scoping Review
Source: Neurotrauma Rep. 2024 May 29;5(1):483–96. doi: 10.1089/neur.2023.0115 (PMC11257139; doi:10.1089/neur.2023.0115)
Supplement: Supplemental Appendix S1 [file neur.2023.0115_supplementalmaterial.docx]

Supplementary Appendices

Associations Between Intracranial Pressure Extremes and Continuous Metrics of Cerebrovascular Pressure Reactivity in Acute Traumatic Neural Injury: A Scoping Review

Kevin Y. Stein,^1^ Fiorella Amenta,^2^ Logan Froese,^1^ Alwyn Gomez,^3,4^ Amanjyot Singh Sainbhi,^1^ Nuray Vakitbilir,^1^ Younis Ibrahim,^3^ Abrar Islam,^1^ Tobias Bergmann,^2^ Izabella Marquez,^2^ and Frederick A. Zeiler^1,3,5,6^

1. Biomedical Engineering, Price Faculty of Engineering, University of Manitoba, Winnipeg, Canada
2. Undergraduate Engineering, Price Faculty of Engineering, University of Manitoba, Winnipeg, Canada
3. Section of Neurosurgery, Department of Surgery, Rady Faculty of Health Sciences, University of Manitoba, Winnipeg, Canada
4. Department of Human Anatomy and Cell Science, Rady Faculty of Health Sciences, University of Manitoba, Winnipeg, Canada
5. Department of Clinical Neuroscience, Karolinska Institutet, Stockholm, Sweden
6. Division of Anaesthesia, Department of Medicine, Addenbrooke’s Hospital, University of Cambridge, Cambridge, UK

**Corresponding Author:**

Kevin Y. Stein BSc

Biomedical Engineering

Faculty of Engineering

University of Manitoba

Winnipeg, MB, Canada

Email: [steink34@myumanitoba.ca](mailto:steink34@myumanitoba.ca)

OrcID: https://orcid.org/0000-0002-5983-008X

**Appendix A: PRISMA Checklist**


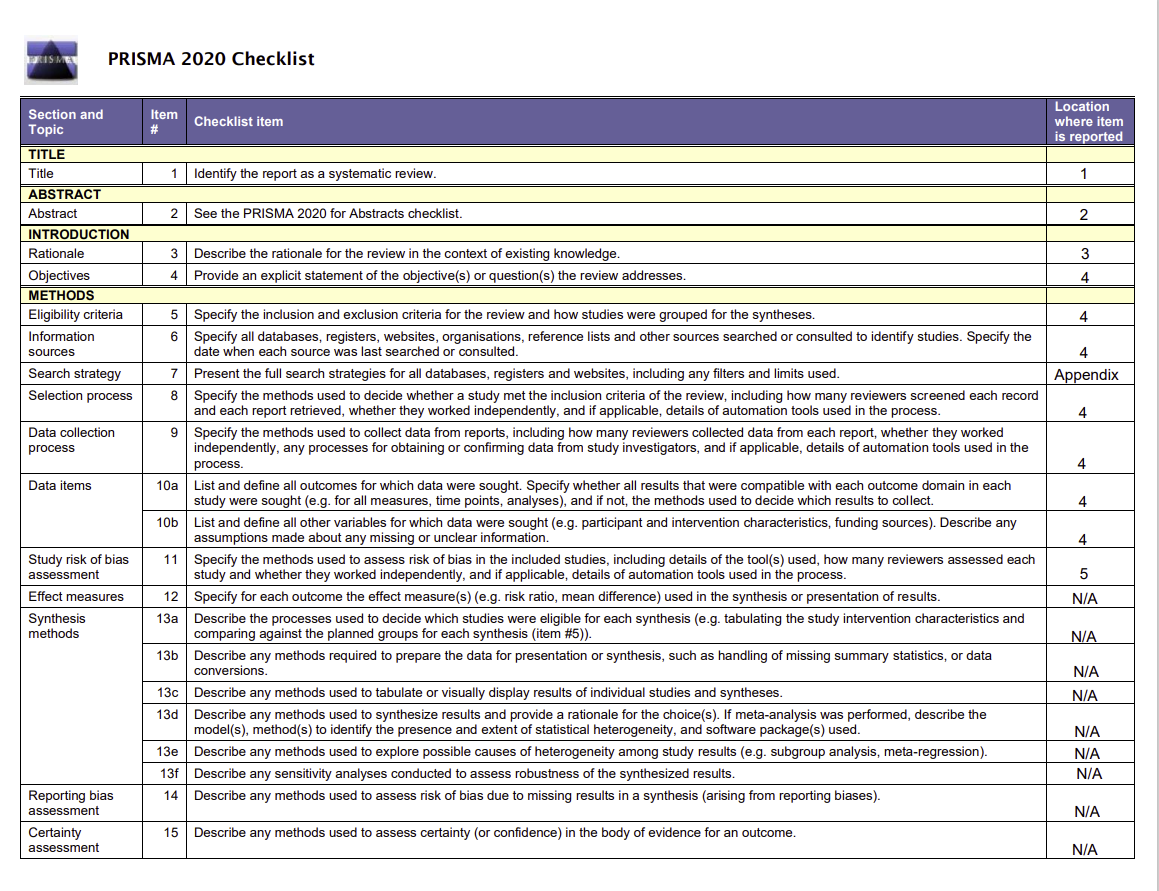


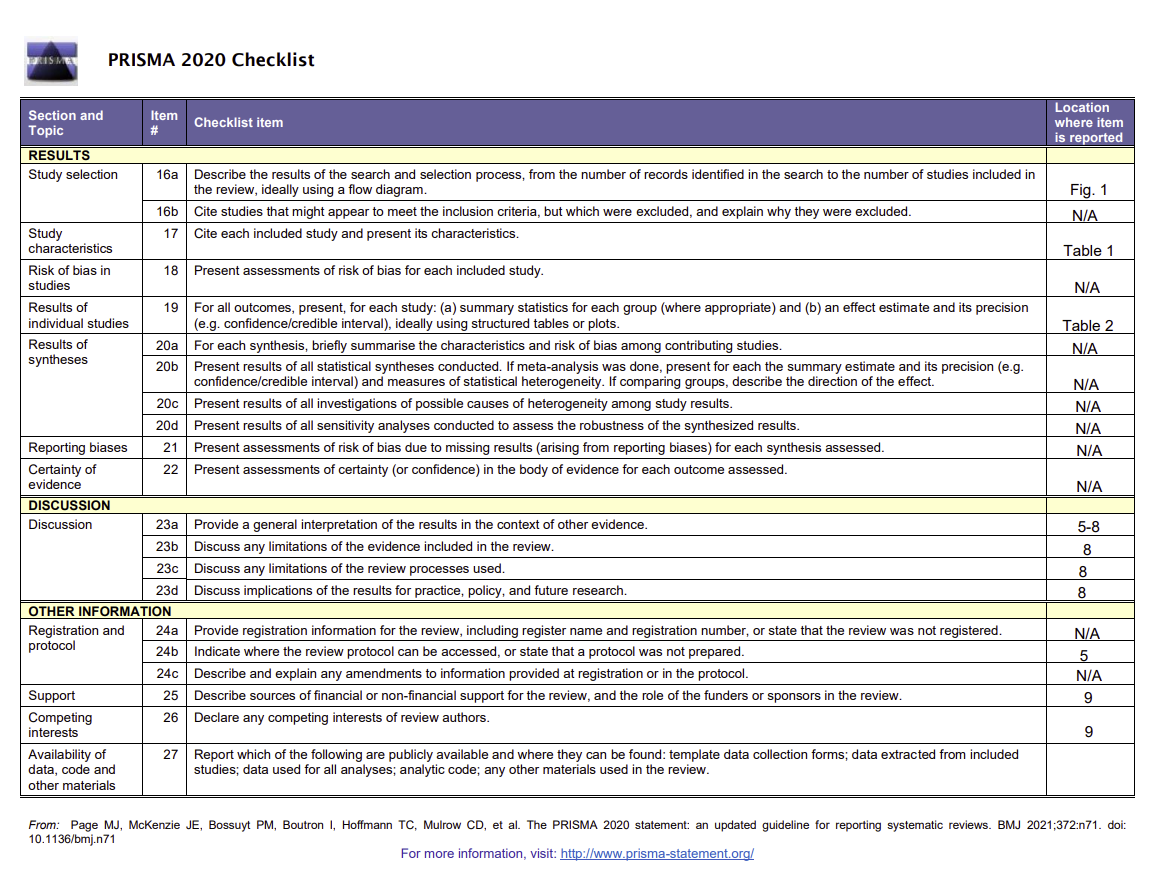


**Appendix B: Sample MEDLINE Search Strategy**

1. traumatic brain injury.mp.
2. TBI.mp.
3. brain injury.mp.
4. cerebral injury.mp.
5. head injury.mp.
6. brain trauma.mp.
7. cerebral trauma.mp.
8. head trauma.mp.
9. craniocerebral trauma.mp.
10. traumatic encephalopathy.mp.
11. neurotrauma.mp.
12. diffuse axonal injury.mp.
13. concussion.mp.
14. 1 OR 2 OR 3 OR 4 OR 5 OR 6 OR 7 OR 8 OR 9 OR 10 OR 11 OR 12 OR 13
15. ICP.mp.
16. intracranial pressure.mp.
17. 15 OR 16
18. Autoregulation
19. Cerebrovascular reactivity
20. Autoregulatory
21. Vasoreactivity
22. Pressure Reactivity Index
23. PRx
24. Pulse Amplitude Index
25. PAx
26. RAC
27. Cerebral Blood Flow Index
28. CBFx
29. CBFx-a
30. Cerebral Oximetry Index
31. Cox
32. COx-a
33. Diastolic Flow Index
34. Dx
35. Dx-a
36. Deoxyhemoglobin Index
37. Hbx
38. Hbx-a
39. Oxyhemoglobin Index
40. HbOx
41. HbOx-a
42. Hemoglobin Volume Index
43. HVx
44. Induced Pressure Reactivity Index
45. iPRx
46. Long Pressure Reactivity Index
47. L-PRx
48. Low-Frequency Autoregulation Index
49. LAx
50. Laser-Doppler Index
51. LDx
52. Lx
53. Lx-a
54. Mean Flow Index
55. Mx
56. Mx-a
57. Oxygen Reactivity Index
58. ORx
59. PRx55-15
60. Systolic Flow Index
61. Sx
62. Sx-a
63. Total Hemoglobin Index
64. THx
65. THx-a
66. Tissue oxygen index
67. Tox
68. TOx-a
69. Wavelet Cerebral Oximetry Index
70. wCOx
71. Wavelet Hemoglobin Volume Index
72. wHVx
73. Wavelet Pressure Reactivity Index
74. wPRx
75. 18 OR 19 OR 20 OR 21 OR 22 OR 23 OR 24 OR 25 OR 26 OR 27 OR 28 OR 29 OR 30 OR 31 OR 32 OR 33 OR 34 OR 35 OR 36 OR 37 OR 38 OR 39 OR 40 OR 41 OR 42 OR 43 OR 44 OR 45 OR 46 OR 47 OR 48 OR 49 OR 50 OR 51 OR 52 OR 53 OR 54 OR 55 OR 56 OR 57 OR 58 OR 59 OR 60 OR 61 OR 62 OR 63 OR 64 OR 65 OR 66 OR 67 OR 68 OR 69 OR 70 OR 71 OR 72 OR 73 OR 74
76. 14 AND 17 AND 75

Mp = title, abstract, original title, name of substance word, subject heading word, floating sub-heading word, keyword heading word, organism supplementary concept word, protocol supplementary concept word, rare disease supplementary concept word, unique identifier, synonyms
